# Development and Evaluation of a Cellular Vehicle-to-Everything Enabled Energy-Efficient Dynamic Routing Application

**DOI:** 10.3390/s23042314

**Published:** 2023-02-19

**Authors:** Mohamed M. G. Farag, Hesham A. Rakha

**Affiliations:** 1 Center for Sustainable Mobility, Virginia Tech Transportation Institute, Blacksburg, VA 24061, USA; 2 College of Computing and Information Technology, Arab Academy for Science, Technology, and Maritime Transport, Alexandria 21500, Egypt; 3 Charles E. Via, Jr. Department of Civil and Environmental Engineering, Virginia Tech, Blacksburg, VA 24061, USA

**Keywords:** C-V2X, eco-routing, ITS, CAV, VANET, smart cities, environmental applications, vehicular networks, V2V, V2I

## Abstract

Cellular vehicle-to-everything (C-V2X) is a communication technology that supports various safety, mobility, and environmental applications, given its higher reliability properties compared to other communication technologies. The performance of these C-V2X-enabled intelligent transportation system (ITS) applications is affected by the performance of the C-V2X communication technology (mainly packet loss). Similarly, the performance of the C-V2X communication is dependent on the vehicular traffic density which is affected by the traffic mobility patterns and vehicle routing strategies. Consequently, it is critical to develop a tool that can simulate, analyze, and evaluate the mutual interactions of the transportation and communication systems at the application level to quantify the benefits of C-V2X-enabled ITS applications realistically. In this paper, we demonstrate the benefits gained when using C-V2X Vehicle-to-Infrastructure (V2I) communication technology in an energy-efficient dynamic routing application. Specifically, we develop a Connected Energy-Efficient Dynamic Routing (C-EEDR) application using C-V2X as a communication medium in an integrated vehicular traffic and communication simulator (INTEGRATION). The results demonstrate that the C-EEDR application achieves fuel savings of up to 16.6% and 14.7% in the IDEAL and C-V2X communication cases, respectively, for a peak hour demand on the downtown Los Angeles network considering a 50% level of market penetration of connected vehicles. The results demonstrate that the fuel savings increase with increasing levels of market penetration at lower traffic demand levels (25% and 50% the peak demand). At higher traffic demand levels (75% and 100%), the fuel savings increase with increasing levels of market penetration with maximum benefits at a 50% market penetration rate. Although the communication system is affected by the high density of vehicles at the high traffic demand levels (75% and 100% the peak demand), the C-EEDR application manages to perform reliably, producing system-wide fuel consumption savings.The C-EEDR application achieves fuel savings of 15.2% and 11.7% for the IDEAL communication and 14% and 9% for the C-V2X communication at the 75% and 100% market penetration rates, respectively. Finally, the paper demonstrates that the C-V2X communication constraints only affect the performance of the C-EEDR application at the full demand level when the market penetration of the connected vehicles exceeds 25%. This degradation, however, is minimal (less than a 2.5% reduction in fuel savings).

## 1. Introduction

By providing real-time, highly reliable, and actionable information flows, cellular vehicle-to-everything (C-V2X) seeks to redefine transportation by enabling better informed decisions. C-V2X can enhance road safety, increase traffic flow efficiency, reduce vehicular environmental impacts, and provide travelers with new valuable communication services. C-V2X will serve as the platform to V2X providing 360° non-line-of-sight awareness and a higher level of predictability for enhanced road safety and autonomous driving. C-V2X technology could be the key that can finally unlock the promise of intelligent transportation systems. C-V2X systems are based on the widely deployed 3GPP LTE cellular specifications [1]. Future communication models will take advantage of newer 5G technology to add features and to improve data rates and latency.

There are two research objectives of this work. The first objective is to assess the environmental effects of a wide deployment of a C-V2X-enabled dynamic routing application. More specifically, we attempt to quantitatively determine how specific C-V2X-enabled applications can reduce CO_2_ emissions and fuel consumption. This analysis will help various stakeholders improve their understanding of the societal benefits of C-V2X, and thereby accelerate the deployment of C-V2X across the US. The second objective is to study the impact of C-V2X communication on the performance of transportation applications.

The proposed methodology entails developing and using a microscopic integrated traffic and C-V2X simulation tool to quantify the network-wide efficiency and environmental impacts of a Connected Energy-Efficient Dynamic Routing (C-EEDR) application. The C-EEDR application is an ITS application that attempts to minimize the vehicle’s fuel consumption levels, by routing vehicles through the most environmentally friendly routes. It utilizes connected vehicle technology to collect real-time fuel consumption information from probe vehicles to compute the best routes. The C-EEDR navigation system assumes the capability of some vehicles (known as sensor vehicles or probe vehicles) to compute the fuel consumption on each traversed road segment and report the computed fuel consumption and the associated road segments to the cloud. The optimal routes are calculated at the cloud and reported back to all the connected vehicles.

**Figure 1:**
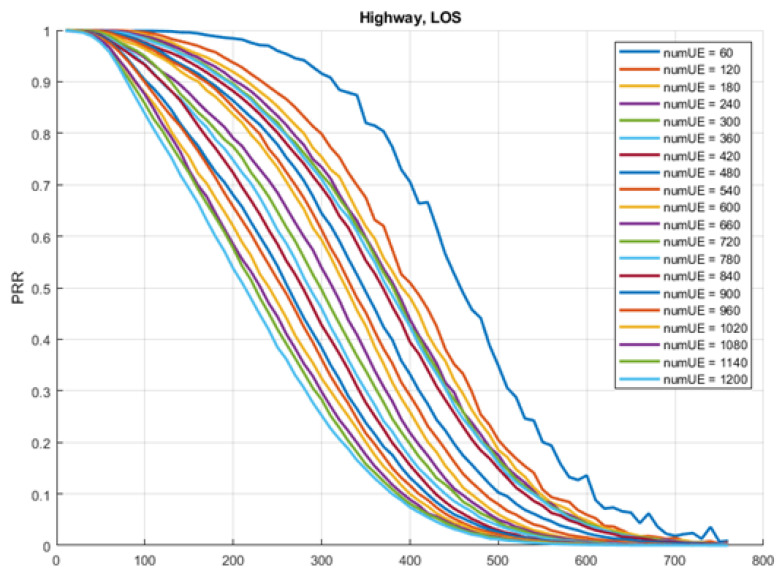
PRR of C-V2X communication data generated using a proprietary simulation software.

Our contributions are:

- We developed a fully integrated traffic and C-V2X modeling framework and used this modeling framework to quantify the system-wide efficiency, energy, and environmental impacts of the C-EEDR application. The evaluation was conducted on a calibrated network of downtown Los Angeles (Figure 1), considering a peak hour of the weekday morning peak period (approximately 144,000 vehicles were modeled). The application testing considered different traffic demand levels and different levels of the market penetration of connected vehicles.
- Our modeling framework also captures the bidirectional interaction of the communication and transportation systems. The traffic density affects the communication network performance. On the other hand, in the C-EEDR navigation application, the traffic mobility is directly affected by the performance of the communication system (i.e., packet reception rate, PRR). A low packet reception rate means reduced information for the application, leading to calculating sub-optimal routes, and resulting in higher fuel consumption levels.
- The developed framework allows us to study and model such complex and challenging systems by capturing the mutual interactions between the communication and transportation systems [2] at a city-level road network scale.
- We compare the C-EEDR application performance in two main cases:
  - The IDEAL communication case, which assumes a perfect communication performance (i.e., no packet loss or delay); and
  - The realistic communication modeling case, in which we utilize a C-V2X communication effectiveness model and study its impact on the system performance.

This paper is organized as follows. Section 2 summarizes the related work regarding energy-efficient dynamic routing. Section 3 gives an overview of the Connected Energy-Efficient Dynamic Routing (C-EEDR) application and its related work. Section 3 describes the implementation and operation of C-EEDR, assuming IDEAL communication. In Section 4, the C-V2X communication model is presented and the operation of the C-EEDR with the communication modeling is described. The simulation network and results are presented in Section 5. Conclusions and future work are described in Section 6.

## 2. Related Work

A previous study [3] developed a stochastic energy-efficient feedback routing system that builds on vehicle connectivity. This system selects vehicle routes based on the the energy consumption experiences of other vehicles within the same vehicle class. All drivers within a specific class are divided into five sub-populations, each comprising 20% of all drivers. The paths for each of these sub-populations are then updated every *t* seconds during the simulation based on real-time measurements of link (arc) energy usage for the specific vehicle class under consideration. The minimum path updates for each vehicle sub-population are staggered in time to avoid situations in which all vehicles select identical paths if the minimum and next minimum path are similar. This logic results in 20% of the driver paths being updated every t/5 s. The selection of the next link that a vehicle should take is made using a vehicle-specific array that lists the entire sequence of links from a vehicle’s current link to its destination. Upon the completion of any link, a vehicle simply submits its energy consumption on the link and then queries this array to determine which link it should utilize next to reach its ultimate destination in the most efficient manner. The vehicle only uses the experiences of other vehicles in the same class to update the energy consumption estimates on a link. This allows for a routing system that is multi-class (up to five classes; vehicles are only affected by experiences of other vehicles in the same class), stochastic (the system can introduce white noise in the link-specific cost function; the user specifies the coefficient of variation of the white noise distribution), and dynamic (based on the updating frequency specified by the user). The authors showed how the benefits varied between the Columbus and Cleveland road networks using the “IDEAL” communication framework. Based on those findings, we expect our proposed system “Connected-Energy Efficient Dynamic Routing (C-EEDR)” application to perform well on other urban networks.

The routing algorithm was further enhanced by developing an ant colony-based eco-routing technique (ACO-ECO) that reduces data latency (the time taken to obtain the link cost information) [4]. The ACO-ECO algorithm is also able to capture randomness in route selection, pheromone updating, and pheromone evaporation. The ACO-ECO algorithm reduced the network-wide fuel consumption and CO_2_ emission levels by 2.3% to 6.0% relative to the feedback eco-routing system. Further enhancements were made using a linear programming technique to compute system-optimum routings [5]. This was the first system optimum eco-routing model that uses linear programming and stochastic route assignment to minimize the system-wide fuel consumption. The system optimum eco-routing reduced the fuel consumption by about 36%, compared to the user equilibrium model.

This energy-efficient routing algorithm was first developed in [6] to select the most energy efficient route, thus minimizing CO_2_ emissions. The algorithm was applied on the road network of the city of Lund, Sweden. This routing algorithm produced a 4% average saving in fuel consumption.

Eco-routing finds the optimal routes based on the fuel consumption cost only. Authors in [7,8] used the travel time and fuel consumption as a multi-objective for finding the best routes for connected and automated vehicles.

The authors in [9] applied the concept of fuel savings not only in the routing context but also in the eco-driving application in the same road network simulations. Two ITS applications were activated in the simulations, namely, eco-driving and eco-routing. The application optimizes the routes for better fuel savings and also optimizes the speed profile to achieve better fuel consumption levels.

The authors in [10] developed a fuel efficient routing algorithm that computes the most fuel-saving route, taking into consideration the detailed mobility profiles of different road segments, the driving behavior, and the different vehicle types. The authors utilized the mobility profiles (including acceleration, deceleration, and idling time) of the road segments instead of assuming an average speed to better estimate the fuel consumption on each road segment. The authors concluded that their approach significantly reduced fuel consumption at the expense of a longer route or more travel time.

The work conducted in [11] explored the effect of including the traffic signals into the dynamic traffic routing problem. The authors devised a 0-1 integer programming approach with a multi-objective function (travel time and energy consumption) to find the energy-efficient route for electric vehicles. The proposed method finds the optimal route that would allow the electric vehicle to go through the road network, avoiding stopping at traffic signals, and thus reducing energy consumption (in decelerating and accelerating). The authors assumed the vehicle receives all the needed information (traffic signal timing) without modeling the communication constraints (i.e., IDEAL communication).

Most of the work conducted in the area of the eco-routing assume an IDEAL communication (i.e., no packet loss or delay) network and does not model its impact on the eco-routing application performance or even the other relation, the impact of the eco-routing application on the communication system performance. As far as we know, there is no work or tool in the literature that studies the mutual bi-directional impact between the communication and the transportation system at both the system performance and the application performance levels.

The authors in [12] assumed wireless connectivity using WiFi. They used a bluetooth-enabled OBD adapter to transmit the fuel consumption data to a phone application on the driver’s cell phone in the car. The application then combined the OBD data with other sensory data and transmits them to an application server upon the availability of WiFi Internet connectivity.

The work in [13] considered the impact of communication on the eco-routing application and used a discrete event simulation to model communication in Vehicular Ad-hoc NETworks (VANET). However, the system was developed for small scale networks and does not scale well with large road networks. In [14], the authors developed a novel analytical model for the Dedicated Short Range Communication (DSRC) communication technology and incorporated it within a microscopic traffic simulator, the INTEGRATION software [2], to enable the modeling of communication and traffic mobility in large-scale transportation systems. In [15], the authors utilized the analytical model for the DSRC communication technology to conduct their sensitivity analysis of both the DSRC communication system and the penetration ratio of connected vehicles and their impacts on the eco-routing application on a city-wide large-scale road network.

The authors in [16] reviewed the studies that analyzed the environmental and safety impact of the vehicle-to-everything (V2X) communication technology on the Intelligent Transportation systems (ITS) applications.

In this work, we utilize industry provided data to model the most recent communication technology, cellular vehicle-to-everything (C-V2X), to analyze the impact of the communication system on the C-EEDR application at different market penetration ratios and different traffic demand levels. We unitize a highly accurate fuel consumption model [17] to estimate the fuel consumption of the vehicles in the road network.

## 3. Energy-Efficient Dynamic Routing Algorithm

The routing application determines the best route the vehicles should take based on a predefined criterion (travel time, distance or fuel consumption). The environmental and economic effect of the transportation system has become an important factor in all decision-making processes. It is of high importance to decrease the fuel/energy consumption of the transportation sector. Energy-efficient routing is a mean of accomplishing this objective where vehicle routes are chosen based on their fuel consumption cost. Fixed routing calculates the best routes based on the given road segments’ cost. The costs used in the fixed routing algorithm could be a historic data of the average cost on the specified road network or could be the cost of travelling with the free flow speed. The traffic mobility patterns are very dynamic and thus require adaptive or dynamic routing algorithms. An important step in dynamic routing is to obtain the real-time periodically updated cost of travelling on a road segment. This requires the vehicles to be able to identify which road segment it is on at any point in time and to be able to communicate the cost of travelling on a road segment. At each of the update intervals, the costs are collected, the routing algorithm is run again using the updated costs, and new routes are produced. The traffic will be assigned the new routes if their costs are better than the old routes.

The routing problem can be modeled as a shortest path problem and is solved using the Dijkstra algorithm [18]. The Dijkstra algorithm is applied periodically with the current road segments’ costs collected from the probe vehicles. In order to prevent the frequent change of routes (oscillating routes), a traffic assignment algorithm is utilized. The routing algorithm produces the top five different best routes. The traffic is randomly distributed between these five routes. Then, the update period is divided into five sub-periods. At each of these sub-periods, the recent costs of the road segments are collected and used to update the current costs. Then, the routes are recalculated using the recent updated costs. In this way, at each updated sub-period, only one fifth of the traffic demand will be updated with the new routes. Another factor that prevents the frequent change of routes is that vehicles do not change their routes unless the new route’s cost is better than the old route’s cost with a predefined threshold.

The fuel consumption of a road segment can be calculated using simplified macroscopic analytical or data-driven models using the average speed on the road segment. A microscopic approach is to use the second-by-second speed and acceleration to calculate the fuel consumption of each vehicle. The microscopic approach provides more accurate calculation but with more computation steps. We adopted the Virginia Tech Comprehensive Power-based Fuel Consumption Model (VT-CPFM) [19] microscopic model to calculate the second-by-second fuel consumption of each vehicle. The details of the fuel consumption model is described in the next section. We used the average fuel consumption of the vehicles that traversed the road segment as a representative of the cost of the fuel consumption on that road segment.

### Fuel Consumption Model

The vehicle’s instantaneous fuel consumption is calculated using the VT-CPFM model. The VT-CPFM is a two-regime model and models the fuel consumption as a second-order polynomial function of the vehicle’s power. The second-order model prevents the occurrence of the bang-bang control when applying the model. Furthermore, a higher-order model cannot be calibrated using publicly available data. Consequently, a second-order model achieves a good trade-off between model accuracy and applicability. In this study, only VT-CPFM-1 is utilized given that VT-CPFM-2 requires engine gear data for model calibration and implementation, which is usually not available. Hence, The VT-CPFM refers to the VT-CPFM-1 model. The VT-CPFM model utilizes the vehicle’s instantaneous power as an input variable. The model parameters can be easily calibrated using publicly available fuel economy data (e.g., Environmental Protection Agency [EPA]-published city and highway gas mileage). Thus, the calibration of the model’s parameters does not require gathering any vehicle-specific field data. The VT-CPFM model equation is:

(1) $$F\left(t\right)=\left\{\begin{array}{cc} a_{0}+a_{1}\;.\;P\left(t\right)+a_{2}\;.\;P{\left(t\right)}^{2}, & \mathrm{if}\;P(t)\geq 0\\ a_{0}, & \mathrm{otherwise} \end{array}\right.$$

where *F*(*t*) is the fuel consumption of a vehicle at time *t*, $a_{0}$, $a_{1}$, $a_{2}$ are the model parameters, *P*(*t*) is the power at time *t*. The vehicle’s power is calculated using the following equation:

(2) $$P\left(t\right)=\left(m\;.\;a\left(t\right)+m\;.\;g\;.\frac{C_{r}}{1000}\left(c_{1}\;.\;v\left(t\right)+c_{2}\right)+0.5\;.\;\rho_{air}\;.\;A_{f}\;.\;C_{d}\;.\;v{\left(t\right)}^{2}+m\;.\;g\;.\;\theta \right)\;.\;v\left(t\right)$$

where *v(t)* is the speed of the vehicle, *a(t)* is the acceleration of the vehicle, *m* is the mass of the vehicle, *g* is the gravitational acceleration, θ is the grade of the road segment, $C_{r}$, $c_{1}$, and $c_{2}$ are the rolling resistance parameters (depend on road surface type, road condition, and tire type), $\rho_{air}$ is the air density, $A_{f}$ is the frontal area of the vehicle, and $C_{d}$ is the aerodynamic drag coefficient of the vehicle.

## 4. Energy-Efficient Dynamic Routing Using IDEAL Communication

In this section, we describe the logic of the C-EEDR application in the context of the INTEGRATION [2], since we used it in our simulations for the evaluation. The INTEGRATION software is an agent-based microscopic traffic assignment and simulation software. It is capable of simulating large-scale traffic road networks at a time granularity of 0.1 s. This high time-resolution allows detailed analyses of many traffic theory phenomena, such as acceleration, deceleration, lane-changing, and car following behavior. It computes a number of measures of performance, including delay, stops, fuel consumption, hydrocarbon, carbon monoxide, carbon dioxide, and nitrous oxide emissions, and the crash risk for 14 crash types. The details of the INTEGRATION software can be found in [2].

INTEGRATION, by default, models the C-EEDR application assuming IDEAL connectivity, i.e., all messages transmitted are received without loss or delay. This assumption simplifies the modeling process from the traffic engineering perspectives and provides an upper bound of the expected benefits of the application. The C-EEDR application is developed as a feedback system that assumes the vehicles are connected and equipped with GPS. Moreover, a vehicle is assumed to be capable of calculating the fuel consumption for each road segment it traverses and communicating this information to the central server. In INTEGRATION, the fuel consumption and emission rates of each vehicle are calculated every second, based on instantaneous speed and acceleration.

Each vehicle accumulates this fuel consumption rate on each road segment it travels on. Then, whenever the vehicle exits that road segment, it updates the road segment’s cost. Based on the IDEAL communication assumption, these updates are promptly added to the routing information at the central server in the cloud.

The vehicle route is a sequence of connected road segments. Thus, if a route $R_{i}$ consists of *k* road segments, the total route fuel consumption cost $F_{R_{i}}$ is the summation of the fuel consumption of the constituting road segments, as expressed in the following equation:

(3) $$F_{R_{i}}\left(t\right)=\sum_{j=1}^{k}\;F_{S_{j}}\left(t\right)$$

where $F_{S_{j}}$ is the fuel consumption on the road segment $S_{j}$ at time *t*.

Initially, $F_{S_{j}}$ is computed based on the free-flow speed. Then, this value is updated based on the updates from probe vehicles. Based on the IDEAL communication assumption, whenever a vehicle exits a road segment $S_{j}$, INTEGRATION uses the reported vehicle’s fuel consumption $F_{v}$ on this road segment to update the road segment’s cost $S_{j}$. It uses a smoothing factor α, as shown in Equation (4); a typical value of α in INTEGRATION is 0.2.

(4) $$F_{S_{j}}\left(t+1\right)=\left(1-\alpha \right)\;.\;F_{S_{j}}\left(t\right)+\alpha \;.\;F_{v}$$

where $F_{v}$ is the estimated fuel consumption of vehicle *v* on the road segment $S_{j}$. The fuel consumption cost is communicated to the central server when the vehicle leaves the road segment. Then, the routing algorithm uses all the communicated road segments costs to update the current ones using Equation (4).

## 5. Energy-Efficient Dynamic Routing Using C-V2X Communication

In this section, we describe the changes to the C-EEDR application to accommodate the limitations of using the realistic communication system. First, we describe the communication model used, then how this model is implemented in the INTEGRATION simulation tool.

We used the cellular vehicle-to-everything (C-V2X) 4G LTE-V communication technology to model the communication system. We used a realistic communication simulation model of the C-V2X communication technology and integrated it with the traffic simulator INTEGRATION [2].

We used Packet Reception Ratio (PRR) curves provided from a communication company [20]. The PRR is the probability that a packet will be received by a vehicle using V2V/V2I C-V2X communication at a specific distance and density of vehicles. The company used its proprietary analysis to produce PRR curves at different distances (0 to 760 m with step 10 m) and vehicle densities (60 to 1200 vehicles with step 60 vehicles). Figure 1 shows the PRR curves produced, which represent a realistic approximation to the real world, as the company’s simulator, used to produce the PRR curves, has been calibrated with the real word measurement data [20]. The curves were generated for vehicles moving on a highway with line of sight (i.e., no buildings or obstacles preventing sight). There is a PRR curve for each density index. The density values represent the number of vehicles around the receiving vehicle within 500 m. The x-axis represents the distance between the transmitting and receiving vehicles. The y-axis represents the probability that the transmitted packet will be received. As can be observed in Figure 1, as the density of the vehicles increases, the PRR decreases even at the same distance. The same behavior is observed with the distance, where the PRR value decreases with increasing the distance.

To use these curves in our simulation tool, we could look up a 76 × 10 × 20 table of PRR values at a specific density and distance. If the required density or distance does not exist, then we can use the nearest values and interpolate for the required values. A faster approach is to fit a curve to those values. We fitted the PRR curves with 2 polynomial functions and an exponential function to come up with a formula that produces a PRR value given any distance between the receiving and the transmitting vehicles, and any number of vehicles around the receiving vehicle. Equation (5) shows the function used to fit the PRR curves. A two-step fitting process was performed to fit the PRR data.

(5) $$PRR=\frac{1}{\frac{a_{1}}{a_{2}*\exp\left(\frac{density}{a_{3}}\right)}+poly_{7}\left(density\right)*\exp\frac{distance}{poly_{8}\left(density\right)}},$$

where $a_{1},a_{2},a_{3}$ are parameters calibrated using curve fitting. $poly_{7}$ and $poly_{8}$ are two polynomial functions of the 7th and 8th degrees, respectively. The variables density is the vehicular density and distance is the distance between the transmitting and the receiving vehicles. Equation (6) shows the polynomial function used to calculate $poly_{7}$ and $poly_{8}$ with *n* equal 7 and 8, respectively.

(6) $$poly_{n}\left(density\right)=\sum_{i=0}^{n}p_{i}*density^{i},$$

where $p_{i}$ are the polynomial coefficients and are calculated using curve fitting.

**Figure 2:**
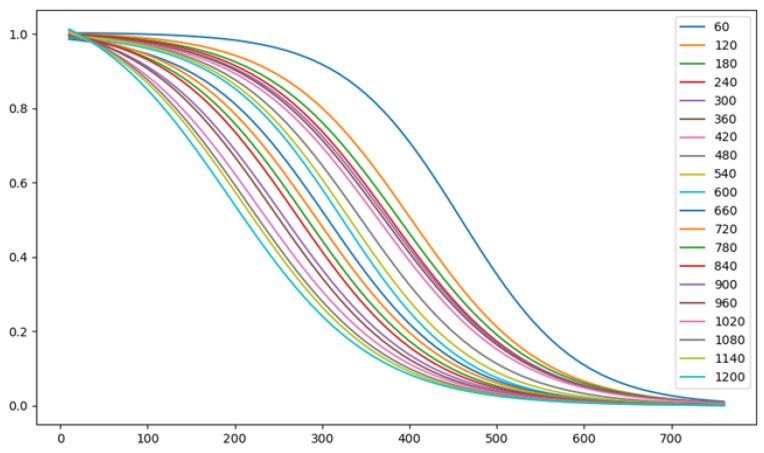
PRR of C-V2X communication fitted the data in the previous figure.

First, we fitted the PRR data (PRR vs. Distance) using an exponential three-parameters function. This fitting step was conducted for each density value (i.e., we had 20 × 3 parameters). Using the fitted function, we can obtain the PRR value for any distance (between 0 and 760 m). Second, we used an exponential, 7th degree polynomial, and 8th degree polynomial functions to fit the three-parameter data produced from the first step. Using these fitted functions, we obtain the corresponding three parameters for any density value (between 60 to 1200 vehicles). Combining all four fitted functions, we obtain the function shown in Equation (5). Figure 2 shows the resulting fitted curves using the PRR data and Equation (5). We can observe that the fitted PRR curves demonstrate the same behavior as the original data.

There are many advantages of using PRR data in this way. First, the data were produced from a communication network simulator that was calibrated using real-world-connected vehicles data, thus providing the most high fidelity and accurate data. Second, this approach allows for running large-scale simulations of hundreds of thousands of vehicles in city-scale road networks in an efficient and reasonable time. This is in contrast to the other approach of using a communication network simulator, where the simulation time is very long due to the huge computation time of the communication network simulators, as it runs at the scale of milliseconds with a high level of details. Similarly, using an analytical communication model can save a huge amount of time but without the high fidelity and accurate results that are produced by a communication network simulator. Thus, our approach combines the advantages of both worlds: the high fidelity of the communication network simulators and the large scale and scalability of the analytical models.

During each time step in the simulation, the traffic simulator identifies for each transmitting vehicle the set of the receiving vehicles and their corresponding distances, as well as the vehicle density around each receiving vehicle in an efficient manner using a spatial index data structure. Using these two pieces of information, we used the PRR Equation (5) to compute the PRR value for each transmitting–receiving vehicle pair. We then draw a random value (0-1), and if the PRR value is bigger than the random value, then the message that contains the fuel consumption cost will be delivered. Otherwise, the message will be lost.

For the C-EEDR application to work correctly, the road segment’s fuel consumption cost needs to be communicated to a central server. Using C-V2X Vehicle-to-Infrastructure (V2I) technology, every vehicle that leaves a road segment transmits its fuel consumption cost on the road segment to the nearest Road-Side Unit (RSU) if there is one. We assume that the RSUs are capable of delivering the information to the central server (using fiber or wireless wide area connectivity). One important factor for the C-EEDR application is the placement of the RSUs. We chose to place the RSUs at the traffic signals to minimize the infrastructure cost. We modeled the problem of choosing the subset of the traffic signals as a set cover problem [21] and used a greedy algorithm to find the minimum number of traffic signals that maximize the coverage of all the traffic signals in the road network within a 500 m communication range. By placing the RSUs at the selected traffic signals, we guarantee that most of the road network will be covered by these RSUs and thus most of the vehicles will be able to transmit their information to the central server through one of these RSUs during their trip anywhere in the road network.

## 6. Simulation Setup

### Simulation Network and Traffic Calibration

**Figure 3:**
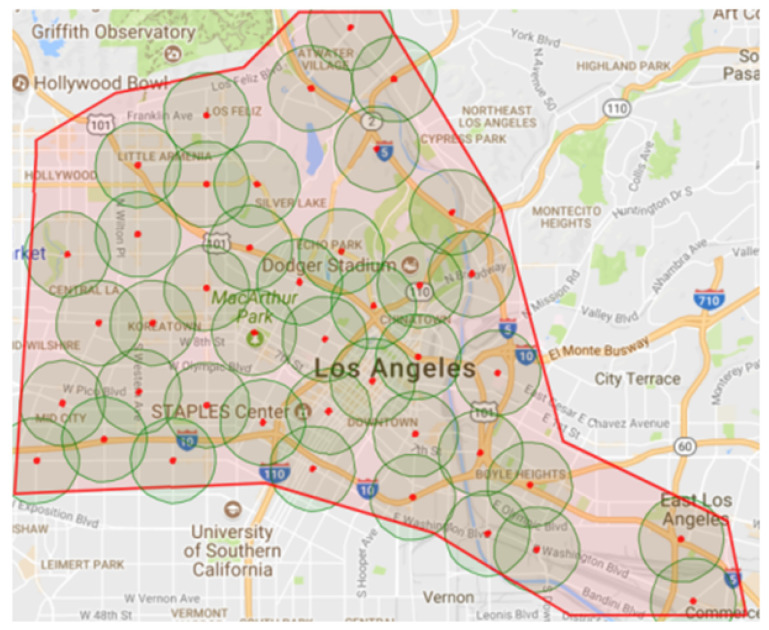
Los Angeles Downtown Road Network.

The downtown area in the city of Los Angeles (LA), shown in Figure 3, is used for the simulation and evaluation. The red points and surrounding circles are the RSU locations and their communication ranges, which are used in communication modeling. This road network is about 133 km${}^{2}$. It has 1625 road network nodes, 3561 road links, and 459 traffic signals. The road network’s vehicle traffic demand was calibrated based on the vehicle count data from loop detectors in the same area. These data are collected from multiple sources, as described in detail in [22]. This traffic demand represents the morning peak hour in the downtown area of the city of LA, which continues for 1 h from 7:00 a.m. to 8:00 a.m. The demand runs for one hour. However, we run the simulation for two hours to give the vehicles enough time to finish their trips and leave the road network. The total number of vehicles that are simulated is approximately 144,000 vehicles.

After applying the greedy algorithm on the set of traffic signals, 126 traffic signals were chosen by the algorithm to cover all the 459 traffic signals. We assume RSUs will be installed at the traffic signals controlling these intersections. This algorithm does not guarantee coverage of the whole road network. However, it covers the maximum number of signalized intersections with a minimum number of RSUs.

## 7. Results

To study the impact of different traffic origin-destination demand (OD) levels, the calibrated traffic rates are multiplied by OD Scaling Factors (ODSFs) ranging from 0.25 through 1.0 at a 0.25 increment, which produces four traffic demand levels (0.25, 0.50, 0.75, 1). For each of these four traffic demand levels, we used nine different market penetration rates of the probe-connected vehicles, (1,2,5,10,20,25,50,75,100). Thus, we ran 36 scenarios using the IDEAL communication configuration, and then reran the same 36 scenarios with the realistic C-V2X V2I communication modeling. The next subsections present and analyze the results at the different traffic demand levels.

We evaluate the C-EEDR application using the IDEAL and C-V2X communication using the following measures: the average fuel consumption, the average travel time, and the average stop delay per vehicle.

### 7.1. Traffic Demand Scale 1

**Figure 4:**
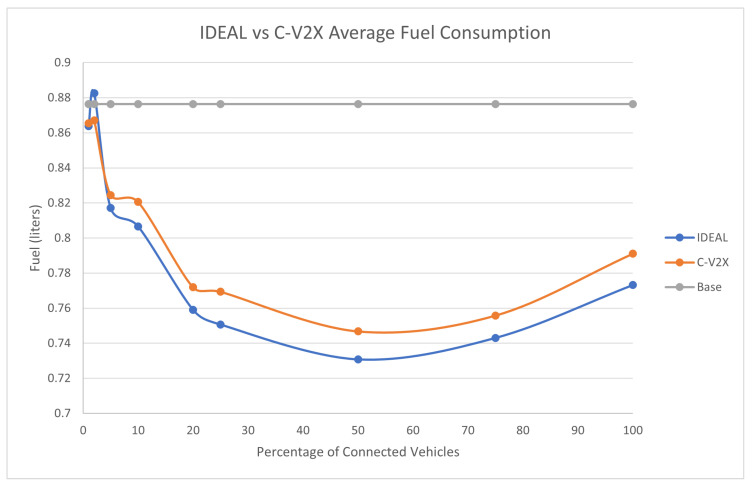
Average network-wide Fuel consumption of Energy-Efficient dynamic routing application using IDEAL and C-V2X communication on LA road network with traffic demand of scale factor 1.

The full traffic demand produced 143,815 vehicles during the first hour of the simulation. Figure 4 compares the fuel consumption of the C-EEDR application using IDEAL and C-V2X communication and contrasts them against the fuel consumption without using the C-EEDR application (base). The base fuel consumption is the average fuel consumption of all the vehicles that travelled through the road network based on the Frank-Wolfe [23] user-equilibrium traffic assignment for the entire simulation period. The C-EEDR application in general helped in decreasing the fuel consumption with the increasing market penetration rates. As more of the vehicles are connected and act as probe vehicles, more information are collected and thus more accurate routing decisions are made. The C-V2X C-EEDR application behavior follows the same behavior as the IDEAL C-EEDR application with slight higher fuel consumption due to the loss of information from the communication system. The fuel consumption increases at higher market penetration rates (>75%).

**Figure 5:**
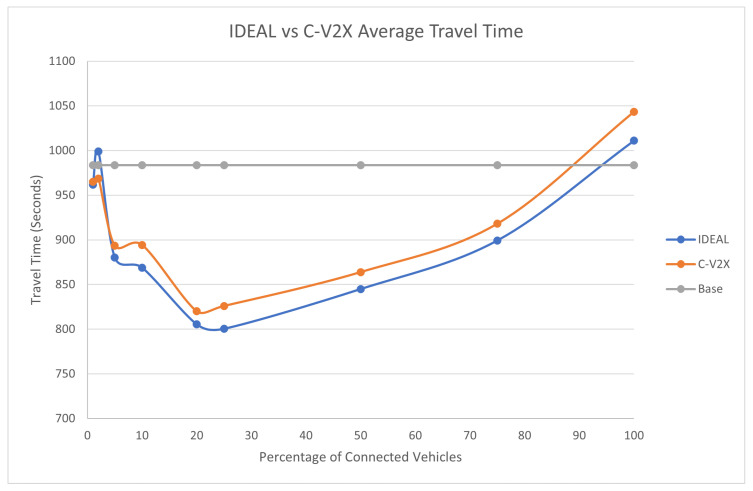
Average network-wide Travel Time of Energy-Efficient dynamic routing application using IDEAL and C-V2X communication on LA road network with traffic demand of scale factor 1.

**Figure 6:**
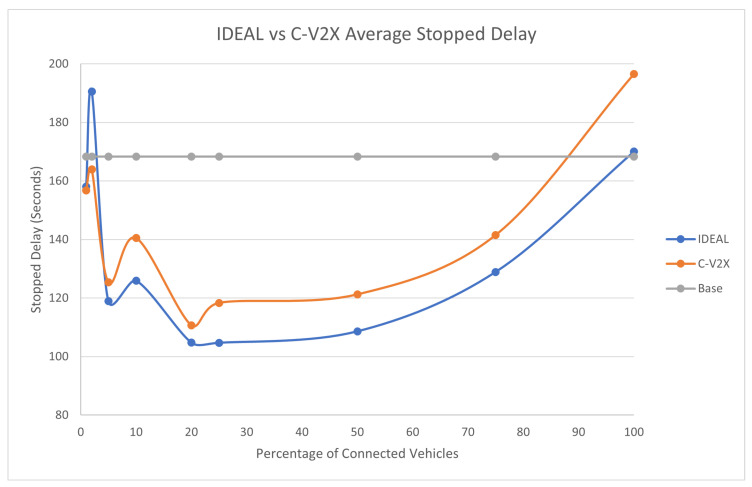
Average network-wide Stopped Delay of Energy-Efficient dynamic routing application using IDEAL and C-V2X communication on LA road network with traffic demand of scale factor 1.

The average travel time and the average stopped delay exhibited a similar behavior as the fuel consumption up to a certain market penetration rate. At the beginning, the travel time decreased as the market penetration rate increased until it reached 25%, and after that, the travel time and stopped delay increased as the market penetration rate increased. The C-V2X showed the same behavior as the IDEAL C-EEDR application, as shown in Figure 5 and Figure 6.

An expected behavior of the C-EEDR application is that when we decrease the average system-wide fuel consumption, we may increase the average travel time and the stopped delay.

It is worth noting that the C-V2X-enabled C-EEDR does not follow the same pattern as the IDEAL C-EEDR with market penetration rates below 10%. This could be due to limitations on the lack of information, which affect the quality of the routing decisions.

We ran the two-tailed Student’s *t*-Test (*p*-values ranged between [0.17–0.82]) between the IDEAL-based C-EEDR and C-V2X-based C-EEDR applications and found that there was no statistically significant difference between the corresponding travel time, stopped delay, and for market penetration rates less than 25% at a significance level of 0.05. For market penetration rates 25% and higher, there was a statistically significant difference between the IDEAL-based C-EEDR and C-V2X-based C-EEDR applications for the corresponding travel time, stopped delay, and the fuel consumption between the mean of the 10 different seed runs. Table 1 shows the percentage change in the fuel consumption, travel time, and stopped delay between the C-V2X and IDEAL-based C-EEDR applications and their corresponding *p*-value. The degradation in performance was less than 3.5% for fuel consumption and travel time and reached 15.6% for the stopped delay.

Although there was a statistical significance between the performance of the C-V2X-based and the IDEAL-based C-EEDR applications at market penetration rates of 25% and higher, we still achieved fuel savings in both cases at all MPR levels. This result confirms the effect of the communication system on the traffic system performance and of the mutual interaction between both systems.

### 7.2. Traffic Demand Scale of 0.75

**Figure 7:**
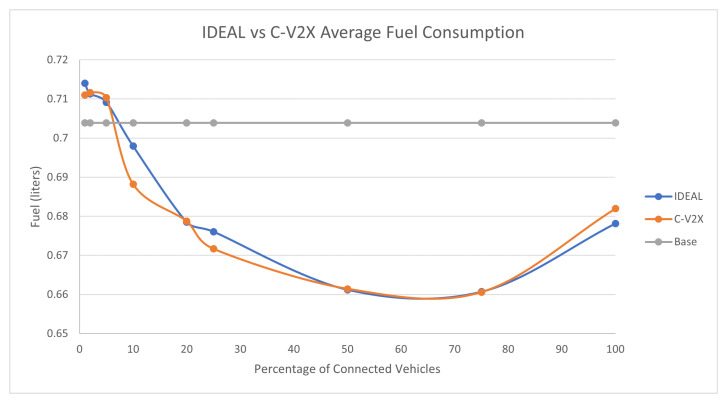
Average network-wide Fuel consumption of Energy-Efficient dynamic routing application using IDEAL and C-V2X communication on LA road network with traffic demand of scale factor 0.75.

**Figure 8:**
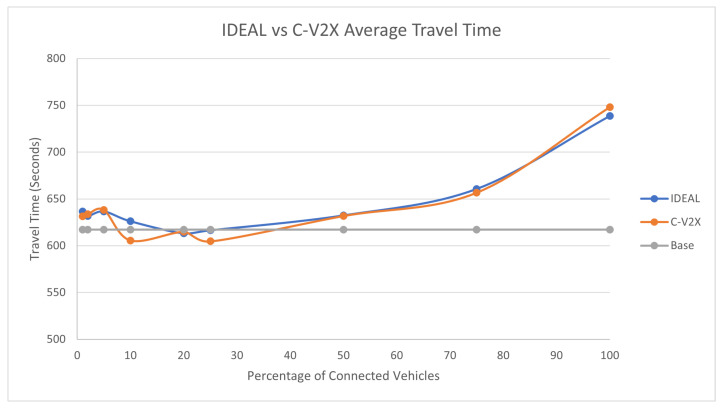
Average network-wide Travel Time of Energy-Efficient dynamic routing application using IDEAL and C-V2X communication on LA road network with traffic demand of scale factor 0.75.

**Figure 9:**
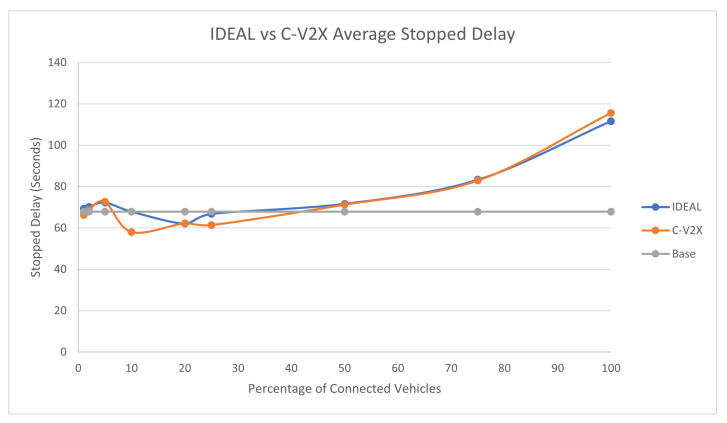
Average network-wide Stopped Delay of Energy-Efficient dynamic routing application using IDEAL and C-V2X communication on LA road network with traffic demand of scale factor 0.75.

The traffic demand at 75% produced 107,960 vehicles during the first hour of the simulation. The C-EEDR application decreased the average system-wide fuel consumption, as shown in Figure 7. We observe the same behavior: as the market penetration rate increases, the fuel consumption decreases. However, the average travel time, as shown in Figure 8, did increase with increasing the market penetration rate, even more than the travel time of the base case. The same behavior is noticed in the case of the stopped delay, as shown in Figure 9.

The behavior of the C-V2X and IDEAL C-EEDR applications are similar at market penetration rates larger than 25% in all the evaluation measures. At market penetration rates less than 25%, the lack of information (due to the low percentage of probe vehicles and packets loss) plays a major role in the quality of the decisions of the C-V2X-based C-EEDR application. We ran the two-tailed *t*-test (*p*-values ranged between [0.17–0.92]) between the IDEAL-based C-EEDR and C-V2X-based C-EEDR applications and found that there was no statistically significant difference between the corresponding travel time, stopped delay, and the fuel consumption between the means of the 10 different seeds for each of the 9 market penetration rates at a significance level of 0.05.

### 7.3. Traffic Demand Scale of 0.50

**Figure 10:**
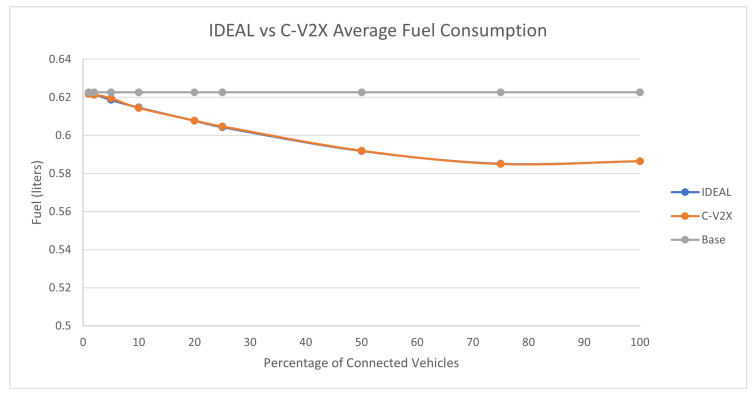
Average network-wide Fuel consumption of Energy-Efficient dynamic routing application using IDEAL and C-V2X communication on LA road network with traffic demand of scale factor 0.5.

A 50% traffic demand produced 70,721 vehicles during the first hour of the simulation. The C-EEDR application decreased the fuel consumption with the increase in the market penetration rate, as shown in Figure 10. Here, we noticed that the C-EEDR application maintained the same behavior (decreasing fuel consumption) even at a full market penetration rate (in contrast with the previous two traffic demand levels).

**Figure 11:**
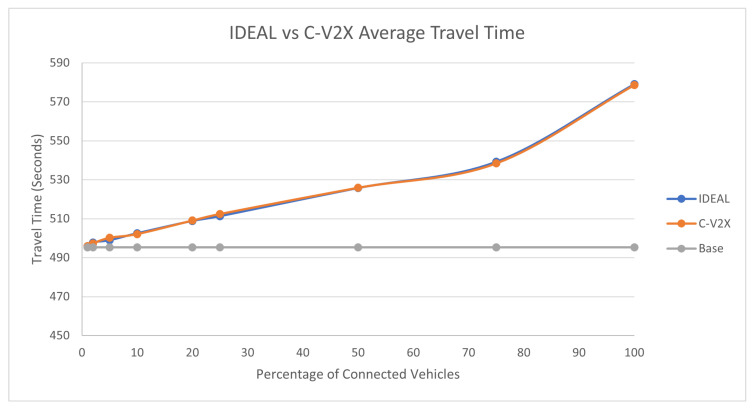
Average network-wide Travel Time of Energy-Efficient dynamic routing application using IDEAL and C-V2X communication on LA road network with traffic demand of scale factor 0.5.

**Figure 12:**
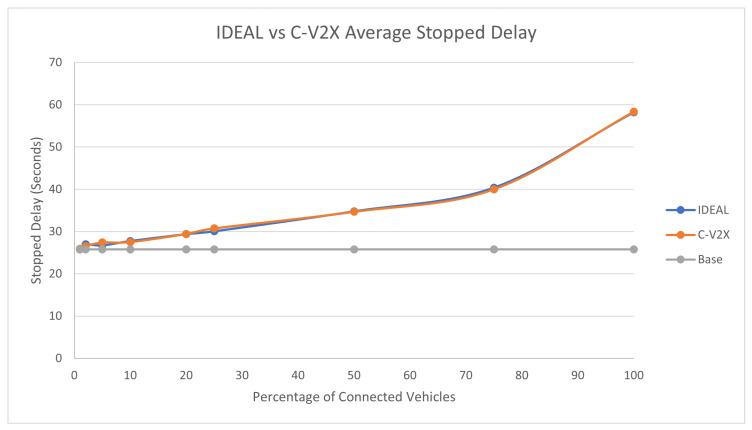
Average network-wide Stopped Delay of Energy-Efficient dynamic routing application using IDEAL and C-V2X communication on LA road network with traffic demand of scale factor 0.5.

However, the system-wide travel time increased as the market penetration rate increased even at low market penetration rates, as shown in Figure 11. The same behavior is noticed also in the stopped delay, as shown in Figure 12.

We ran the two-tailed *t*-test (*p*-values ranged between [0.15–0.86]) between the IDEAL-based C-EEDR and C-V2X-based C-EEDR applications and found that there was no statistically significant difference between the corresponding travel time, stopped delay, and the fuel consumption between the means of the 10 different seeds for each of the 9 market penetration rates at a significance level of 0.05.

### 7.4. Traffic Demand Scale of 0.25

**Figure 13:**
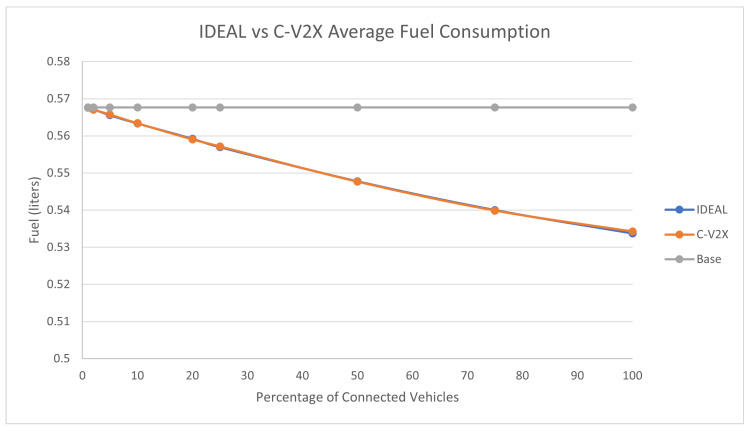
Average network-wide Fuel consumption of Energy-Efficient dynamic routing application using IDEAL and C-V2X communication on LA road network with traffic demand of scale factor 0.25.

**Figure 14:**
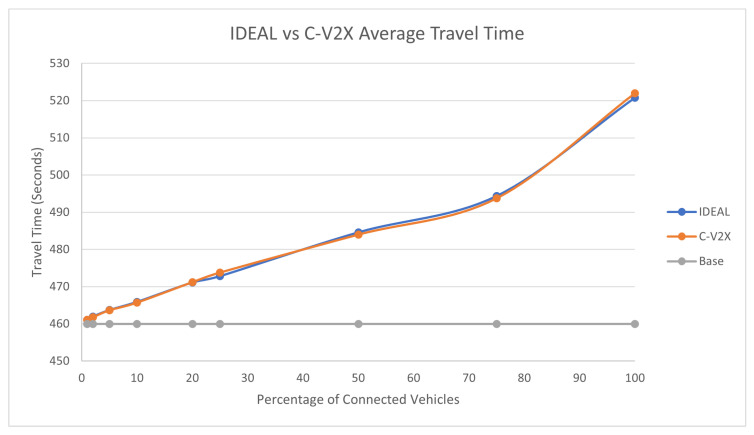
Average network-wide Travel Time of Energy-Efficient dynamic routing application using IDEAL and C-V2X communication on LA road network with traffic demand of scale factor 0.25.

**Figure 15:**
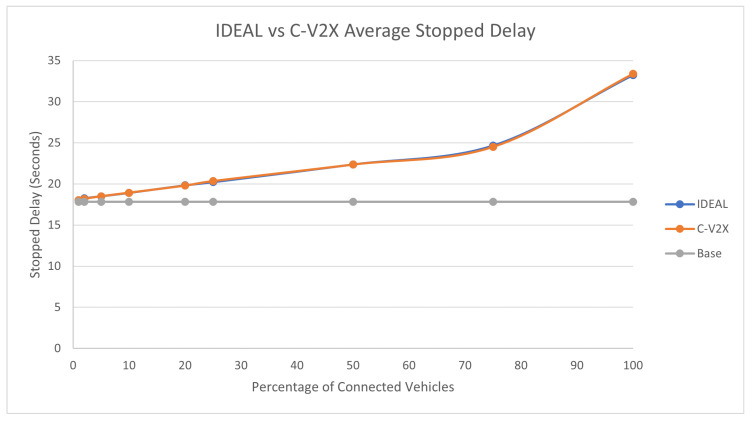
Average network-wide Stopped Delay of Energy-Efficient dynamic routing application using IDEAL and C-V2X communication on LA road network with traffic demand of scale factor 0.25.

The 25% traffic demand produced 34,449 vehicles during the first hour of the simulation. As expected, the C-V2X and IDEAL C-EEDR applications demonstrated the same behavior, as previous traffic demands in terms of the fuel consumption, travel time, and stopped delay, as shown in Figure 13, Figure 14, and Figure 15, respectively.

With low and medium traffic demands (25%, 50%, and 75%) levels, the C-EEDR application exhibits the normal behavior where decreasing the system-wide fuel consumption leads to increasing the system-wide travel time and stopped delay. However, in a high traffic demand level (100% case), the C-EEDR application not only decreased the system-wide fuel consumption but also managed to decrease the system-wide travel time and stopped delay in almost all of the market penetration rates except for the 100% one.

We ran the two-tailed *t*-test (*p*-values ranged between [0.18–0.96]) between the IDEAL-based C-EEDR and C-V2X-based C-EEDR applications and found that there was no statistically significant difference between the corresponding travel time, stopped delay, and the fuel consumption between the means of the 10 different seeds for each of the 9 market penetration rates at a significance level of 0.05.

### 7.5. Fuel Savings

**Figure 16:**
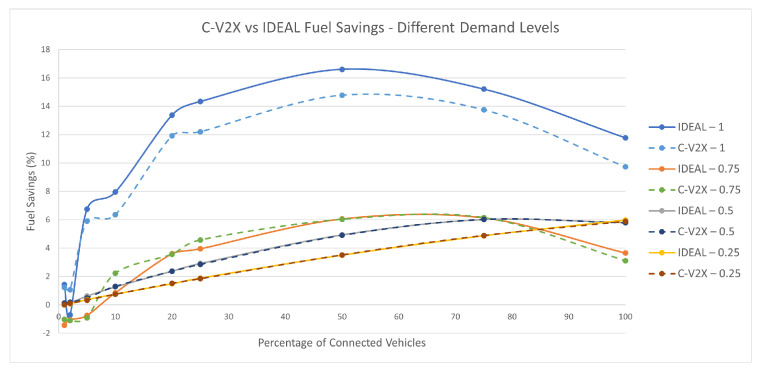
Average network-wide Fuel Savings of Energy-Efficient dynamic routing application using IDEAL and C-V2X communication on LA road network with different traffic demand scale factors.

The maximum fuel savings was achieved (as expected) in the 100% traffic demand with 18% savings at a 50% market penetration rate. At the 75% traffic demand, the maximum fuel savings were 5% at a 75% market penetration rate. The maximum savings at 50% and 25% traffic demands were 6% at the 100% market penetration rate. Figure 16 shows the fuel savings for each of the four traffic demand levels at different market penetration rates.

### 7.6. C-V2X Communication Performance

**Figure 17:**
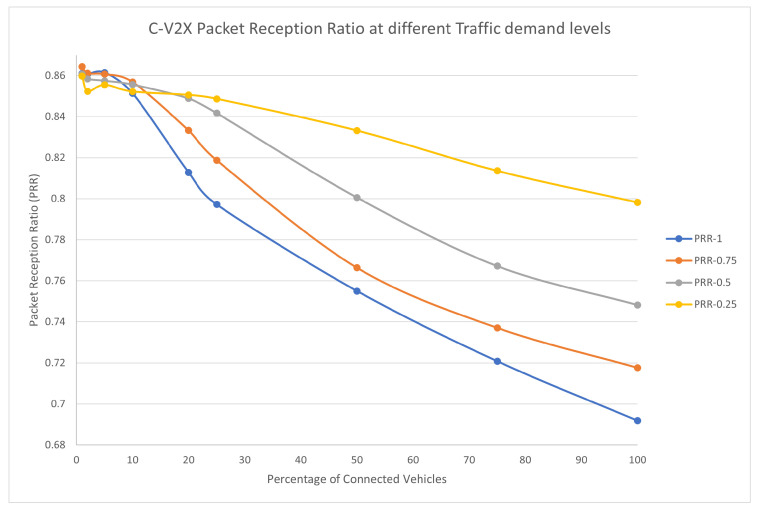
Average Packet Reception Ratio using C-V2X-based Energy-Efficient dynamic routing application in LA road network with different traffic demand scale factors.

To understand the behavior of the C-V2X C-EEDR application, we investigated the performance of the C-V2X communication system. We measured the average PRR value during the whole simulation in all of the simulations we ran. Figure 17 shows the PRR values at different market penetration rates for the four traffic demand levels (25, 50, 75, and 100%, respectively).

There are two observations to note here. First, as the traffic demand increases, the performance of the C-V2X communication system decreases. This is clear as the PRR value decreases with the different traffic demand levels at the same market penetration rate. Second, as the market penetration rate increases, the performance of the C-V2X communication system decreases. It is clear that as more connected probe vehicles enter the system, the traffic density increases and the traffic system becomes more congested, and, consequently, the communication system becomes more congested, which leads to more lost packets.

The C-V2X communication protocol provides reliability to the C-EEDR application with a minimum of 70% of the messages delivered in the case of 100% traffic demand and 100% market penetration and 80% of the messages received in the case of the 25% and 100% market penetration rate.

## 8. Findings and Conclusions

The research presented in this paper studied the impact of C-V2X communication technology on the performance of an Energy-Efficient Dynamic Routing application. We leveraged a communication dataset produced from the industry to model the C-V2X communication system. The data were used to calibrate a general function that produces the Packet Reception Ratio (PRR) given the distance between the transmitting and receiving vehicles and the vehicle density in the vicinity of the receiving vehicle within the communication range. We incorporated the communication model in the INTEGRATION microscopic traffic simulator, which supports large-scale traffic simulations. The existing IDEAL Connected Energy-Efficient Dynamic Routing (C-EEDR) application was modified to incorporate our C-V2X model and account for the C-V2X communication constraints (mainly packet losses). The C-EEDR application was evaluated using the IDEAL communication setting (default implementation without packet loss) and using the C-V2X communication model. The simulations were run on the Los Angeles (LA) downtown large-scale road network considering four traffic demand levels and nine market penetration rates. We also compared the performance of the C-EEDR application to the base case where the application is not enabled. The base case used the Frank–Wolf user equilibrium method for routing the vehicles in the road network.

The simulation results demonstrated that the C-V2X-based C-EEDR application followed the same behavior as the IDEAL-based C-EEDR application with a slight decrease in benefits. The results demonstrate that the C-EEDR application achieves a fuel savings of up to 16.6% and 14.7% in the IDEAL and C-V2X communication cases, respectively, for a peak hour demand on the downtown Los Angeles network considering a 50% level of a market penetration of the connected vehicles. The results demonstrate that the fuel savings increase with the increasing levels of market penetration at lower traffic demand levels (25% and 50% of the peak demand). At higher traffic demand levels (75% and 100%), the fuel savings increase with the increasing levels of market penetration with maximum benefits at a 50% market penetration rate. Although the communication system is affected by the high density of vehicles at the high traffic demand levels (75% and 100% the peak demand), the C-EEDR application manages to perform reliably, producing system-wide fuel consumption savings.The C-EEDR application achieves fuel savings of 15.2% and 11.7% for the IDEAL communication and 14% and 9% for the C-V2X communication at the 75% and 100% market penetration rates, respectively. Finally, the paper demonstrates that the C-V2X communication constraints only affect the performance of the C-EEDR application at the full demand level when the market penetration of the connected vehicles exceeds 25%. This degradation, however, is minimal (less than a 2.5% reduction in fuel savings).

The C-V2X communication protocol demonstrated a reliable performance that allowed the C-EEDR application to achieve benefits even in the highest traffic demand levels (100%) and at full market penetration rates (100%) in contrast to previous studies, where the traffic system failed due to the failure of the underlying communication system used on highly congested transportation networks.

The results demonstrate the importance of the C-EEDR application when applied in highly congested road networks where the application managed to achieve savings not only in fuel consumption, but also in the average system-wide travel time and the stopped delay.

## Figures and Tables

**Table 1:** The percentage change between the C-V2X and IDEAL-based C-EEDR applications performance in terms of Travel Time (TT), Stopped Delay (SD) and fuel consumption.

MPR (%)	TT (%)	SD (%)	FC (%)
1	−0.33 (0.88)	0.84 (0.91)	−0.18 (0.88)
2	3.03 (0.21)	13.95 (0.1)	1.76 (0.18)
5	−1.5 (0.21)	−5.37 (0.25)	−0.9 (0.19)
10	−2.94 (0.08)	−11.6 (0.11)	−1.73 (0.08)
20	−1.82 (0.12)	−5.66 (0.34)	−1.69 (0.02)
25	−3.2 (0.01)	−13.06 (0.01)	−2.49 (0)
50	−2.25 (0)	−11.65 (0)	−2.19 (0)
75	−2.1 (0.01)	−9.81 (0)	−1.72 (0)
100	−3.21 (0)	−15.63 (0)	−2.31 (0)

## Data Availability

Not applicable.

## Abbreviations

The following abbreviations are used in this manuscript:

C-V2X	Cellular vehicle-to-everything
ITS	Intelligent transportation system
V2V	Vehicle-to-vehicle
V2I	Vehicle-to-Infrastructure
C-EEDR	Connected Energy-Efficient Dynamic Routing
